# Effect of β_2_ -adrenergic receptor gene Arg16Gly polymorphisms on response to long-acting β_2_-agonist in Chinese Han asthmatic patients

**DOI:** 10.1186/2049-6958-9-22

**Published:** 2014-04-11

**Authors:** Yuying Qiu, Deping Zhang, Yu Qin, Kai-Sheng Yin

**Affiliations:** 1 Department of Respiratory Medicine, Drum Tower (Gulou) Hospital Affiliated to Nanjing University Medical School, 321 Zhongshan Road, Nanjing 210008, China; 2 Department of Non-Communicable Chronic Disease Control, Jiangsu Provincial Center for Disease Control and Prevention, 172 Jiangsu Road, Nanjing 210009, China; 3 Department of Respiratory Medicine, the First Affiliated Hospital of Nanjing Medical University, 300 Guangzhou Road, Nanjing 210029, China

**Keywords:** Asthma, β_**2**_-adrenergic receptor, Salmeterol, Long-acting β_2_-agonist, Fluticasone propionate, Polymorphism, Genotype

## Abstract

**Background:**

To evaluate the effect of variation of the Arg16Gly polymorphism of the β_2_-adrenergic receptor gene on clinical response to salmeterol administered with fluticasone propionate in Chinese Han asthmatic patients.

**Methods:**

Moderate persistent asthmatic patients (n = 62) currently receiving short-acting β_2_-agonists were administered twice-daily therapy with salmeterol/fluticasone propionate 50/250 μg in a single inhaler for 12 weeks, followed by a 2-to-4-day run-out period. Using direct DNA sequencing, five single nucleotide polymorphisms (SNPs) in the promoter and coding block regions of β_2_-adrenergic receptor gene were determined in 62 subjects and haplotypes were combined.

**Results:**

There was sustained and significant improvement (p < 0.001) over baseline in all measures of asthma control in subjects receiving salmeterol and fluticasone, regardless of Arg16Gly genotype. However, there was no significant difference in the improvement among three genotypes (p > 0.05). Responses to salmeterol did not appear to be modified by haplotype pairs (p > 0.05). During the run-out period, all subjects had similar decreases in measures of asthma control, with no differences between genotypes (p > 0.05).

**Conclusions:**

Response to salmeterol does not vary with Arg16Gly polymorphisms after chronic dosing with inhaled corticosteroids in Chinese Han asthmatic patients.

## Background

Beta_2_-adrenergic receptor gene (ADRB2) is an intronless gene located on chromosome 5q31.32 [1]. Screens of ADRB2 have revealed at least 19 single nucleotide polymorphisms (SNP) within the coding and promoter region, some of which might influence response to β_2_-agonists [2-4]. The most prominent coding SNP is characterized by substitution of glycine for arginine at codon 16 (Arg16Gly), which occurs 38.3% in the Chinese Han asthmatic patients [5].

Asthmatic patients who are homozygous for Arg/Arg at the 16^th^ amino acid position benefit less from treatment with long-acting β_2_-agonists (LABA) and inhaled corticosteroids (ICS) than do those homozygous for Gly/Gly in other asthmatic population [6].

To elucidate whether there is a genotype-dependent influence of regular use of LABA combined with ICS in Chinese Han asthmatic patients, we conducted a 12-week trial of combined fluticasone propionate 250 μg and salmeterol 50 μg in moderate persistent asthmatic patients, and compared clinical outcomes according to the ADRB2 genotypes/haplotypes.

## Methods

Subjects aged 18 years or older and with a history of persistent asthma of at least 6 months were included in the study, as describe before [5]. The study protocols were reviewed and approved by the Review Board of Drum Tower Hospital Affiliated to Nanjing University Medical School.

Patients with moderately persistent asthma were diagnosed based on the Global Initiative for Asthma (GINA) guidelines by Drum Tower Hospital Affiliated to Nanjing University Medical School, and recruited in our study. Eligible subjects entered a 2-week run-in period, and all participants replaced their oral or inhaled SABAs with albuterol prescribed as needed for the relief of acute asthma symptoms. Peak expiratory flow (PEF), albuterol use, asthma symptoms, and nighttime awakenings were recorded daily by the subjects on a diary card. After the run-in period, participants meeting randomization criteria entered the study and received fluticasone propionate/salmeterol (FSC), 250/50 μg diskus twice daily. The criteria included the best FEV_1_ between 60% and 80% of predicted value; the best predose FEV_1_ within ±12% obtained at screening; 5 or more days requiring albuterol use or a diary card asthma symptom score of ≥2 on 3 or more days by using a 6-point scale (0 = no symptoms, 5 = severe symptoms during the previous week). Sixty-two eligible asthmatic patients were involved in the study. Baseline data for PEF, albuterol use, asthma symptoms, and nighttime awakenings were defined as the mean value over the 7 days before randomization. Baseline FEV_1_ was defined as the randomization visit FEV_1_ measurement. During the study, FEV_1_ was measured at treatment weeks 1, 4, 8, and 12, and 3 days after the treatment. After 12 weeks of treatment with FSC, treatment was discontinued for 2 to 4 days while subjects continued to use albuterol as needed and recorded daily symptoms and PEF on diary cards. Baseline for this run-out period was defined as the average of the last 7 days of the FSC treatment period.

Beta_2_-AR polymorphisms in positions −47, −20, 46, 79, and 252 were determined by direct sequencing of PCR products obtained with the following primers: 5′CAC CAC AGC CGC TGA ATG AGG 3′and 5′GGC TTG GTT CGT GAA GAA GTC 3′. The 710-bp PCR fragments were purified with a commercial kit and sequenced using a fluorescently labeled dye terminators technique in an ABI Prism 310 capillary sequencer (PE Biosystems, Foster City, CA).

Haplotypes were estimated from unphased genotypes by using an extension of Clark’s algorithm [7]. The most common five SNPs haplotypes were distinguished from each other using only two SNPs (46 and 79), which coded for codon 16 and codon 27, respectively. The genotype/haplotype frequencies for each polymorphism were tested for deviation by the Hardy–Weinberg equilibrium.

Data was entered and cleaned in Excel 2003. Variables were presented as means ± standard deviations (SD), with chi-square test for categorical variables and ANOVA for continuous variables. Evaluation of the effect of genotype/haplotypes on each clinical parameter was carried out by ANOVA. All analyses were conducted in SPSS 13.0 statistical software (SPSS Inc., Chicago, IL, USA).

## Results

There were no significant differences in demographic, clinical and pulmonary function characteristics across the Arg16Gly genotypes at baseline (p > 0.05).There was sustained and significant improvement (p < 0.001) compared with baseline values in all measurements of asthma control in all Arg16Gly genotypes. However, the changes of all measurements were not significantly different across genotype subgroups (p > 0.05) (Table 1). No significant differences were identified between haplotype pairs and changes of all the measurements (Table 2). During the run-out phase, no differences were noted in any of the clinical responses to FSC withdrawal across the genotypes (Table 3).

**Table 1:** Changes from baseline in the Arg16Gly genotype at the end of 12 weeks of treatment with FSC

***ADRB2*** **marker**	**Morning PEF (L/min)**	**FEV**_**1**_**(L)**	**Albuterol use (puffs/d)**	**Asthma symptom score**
Arg16Gly genotype				
ArgArg(n = 21)	114.4 ± 21.5	0.44 ± 0.09	−3.81 ± 1.94	−1.10 ± 0.78
ArgGly(n = 26)	103.3 ± 23.7	0.43 ± 0.07	−3.77 ± 1.95	−0.96 ± 0.53
GlyGly(n = 15)	100.3 ± 14.7	0.45 ± 0.11	−3.80 ± 1.86	−0.93 ± 0.59

No significant difference.

**Table 2:** Changes from baseline among five haplotype pairs at the end of 12 weeks of treatment with FSC stratified by haplotype pairs

**Haplotype pair**	**Morning PEF (L/min)**	**FEV**_**1**_**(L)**	**Albuterol use (puffs/d)**	**Asthma symptom score**
AC/AC	108.3 ± 25.1	0.46 ± 0.10	−3.50 ± 2.00	−0.79 ± 0.16
GC/GC	89.0 ± 18.5	0.41 ± 0.09	−3.57 ± 1.90	−1.00 ± 0.23
GC/GG	104.4 ± 31.1	0.49 ± 0.11	−4.60 ± 1.89	−1.20 ± 0.45
GC/AC	111.4 ± 28.1	0.42 ± 0.07	−3.85 ± 2.06	−1.05 ± 0.51
GG/AC	103.7 ± 27.9	0.45 ± 0.05	−4.33 ± 1.63	−1.17 ± 0.75

No significant difference.

**Table 3:** Differences between the last 7 days on treatment and the run-out period stratified by genotype for subjects previously treated with FSC

	**Genotype**		
	**Arg/Arg(n = 21)**	**Arg/Gly(n = 26)**	**Gly/Gly(n = 15)**
Morning PEF (L/min)	−38.2 ± 8.6	−24.2 ± 10.2	−34.3 ± 9.8
FEV_1_ (L)	−0.09 ± 0.05	−0.10 ± 0.03	−0.10 ± 0.03
Total albuterol use	0.71 ± 0.23	0.69 ± 0.38	0.73 ± 0.28
Asthma symptom score	0.43 ± 0.17	0.35 ± 0.19	0.33 ± 0.14

No significant difference.

## Discussion

Our study showed that clinical response to the LABA salmeterol did not differ among ADRB2 Arg16Gly SNPs during chronic dosing in the presence of an ICS. Specifically, there were sustained and quantitatively similar improvements in lung function, symptoms, and albuterol use during chronic treatment, regardless of Arg16Gly genotype. Although not powered to definitively evaluate a haplotype effect, our haploype analysis supports the individual SNP results.

Drysdale et al. [2] showed the lowest response in their 4/4 haplotype, corresponding to our Arg16Gln27/Arg16Gln27 haplotype, with a total of 14 subjects as opposed to the 24 in our analysis. Although we did not measure all 13 possible SNPs, using the 5 SNPs at position −47, −20, 46, 79, and 252, we were able to evaluate the most frequent haplotypes corresponding to those previously reported [2,8].

The results of the study were consistent with the findings of Taylor et al. [9], who demonstrated no differential response in exacerbations or lung function in subjects receiving salmeterol, regardless of genotype. Furthermore, our results supported the finding of Bleecker et al. [10] who suggested that Gly16Arg genotypes might not be a genetic determinant of reduced responses to chronic LABA therapy in the presence of ICS therapy in subjects with asthma. In addition, a retrospective study by Klotsman et al. [11] and a recent 6-month double blind randomized study by Bleecker et al. [12] revealed no genotype effect of ADRB2 on the combination therapy.

In contrast, Salmeterol or Corticosteroids (SOCS) trial and Salmeterol +/−Inhaled Corticosteroid (SLIC) trials demonstrated a genotypic-difference in LABA response [13,14]. Genotypic analysis of the SOCS and SLIC cohorts by Weschler [6] revealed that Arg16Arg had a lower morning PEF than Gly16Gly during salmeterol therapy. These results supported an ADRB2 genotype effect on responses to LABA therapy. Furthermore, two retrospective studies showed an ADRB2 genotype effect on response to therapy with the β_2_-agonists [15,16]. In addition, our results were different from a Korean study which suggested that the ADRB2 genotype may dictate choice of treatment strategy [17,18].

The inconsistent results could be attributed to various factors, including ethnic difference, sample size, differences in study design, asthma severity, and intrinsic activity of the β_2_-agonist evaluated, linkage disequilibrium and concomitant use of an ICS.

In our study, LABA treatment was combined with ICS, therefore it is quite possible that the differences in response to ICS among genotype groups could account for the observed differences. However, this possibility is quite unlikely, because all the study subjects used the same regimen of ICS during the 2-week run-in, and showed improvement in both FEV_1_ and PEF, with no significant differences observed between genotype groups.

There were some limitations in our study. In the present study the observations did not eliminate completely a genetic interaction. In addition, the haplotype analysis did not reveal any significant influence on response, but the small sample size and the lack of ethnic representation might limit these conclusions.

## Conclusions

In summary, this study evaluating associations between the polymorphic gene encoding for the β-agonist drug target and responses to therapy with LABA showed that response to salmeterol does not vary by ADRB2 genotypes or haplotypes during chronic dosing in the presence of an ICS.

However, larger prospective clinical pharmacogenetic studies are further needed to help elucidate this field.

## Consent

Written informed consent was obtained from the patient’s guardian/parent/next of kin for the publication of this report and any accompanying images.

## Abbreviations

ADRB2: Beta2- β_2_-adrenergic receptor gene; FSC: Fluticasone propionate/salmeterol; FEV1: Forced expiratory volume in one second, ICS, Inhaled corticosteroids; LABAs: Long-acting β_2_-agonists; SNP: Single nucleotide polymorphism.

## Competing interests

There was no conflict of interest among all authors.

## Authors’ contributions

YYQ designed study, interviewed cases and controls, collected samples, data entry, did lab experiments, and wrote up journal article. DPZ instructed study design and lab measurement. YQ managed and analyzed data, review the article. YKS instructed study design and helped liaison at hospitals. All authors read and approved the final manuscript.
